# Histopathological Examination and Endoscopic Sinusectomy: Is It Possible?

**DOI:** 10.3389/fsurg.2022.793858

**Published:** 2022-03-03

**Authors:** Sara Vertaldi, Pietro Anoldo, Grazia Cantore, Alessia Chini, Anna D'Amore, Maria D'Armiento, Nicola Gennarelli, Francesco Maione, Michele Manigrasso, Alessandra Marello, Pietro Schettino, Carmen Sorrentino, Loredana M. Sosa Fernandez, Giovanni D. De Palma, Marco Milone

**Affiliations:** ^1^Department of Clinical Medicine and Surgery, University of Naples “Federico II”, Naples, Italy; ^2^Department of Advanced Biomedical Sciences, University of Naples “Federico II”, Naples, Italy; ^3^Pathology Unit, Department of Public Health, University of Naples “Federico II”, Naples, Italy; ^4^Embryos Fertility Center, Salerno, Italy

**Keywords:** pilonidal disease, EPSiT, malignant degeneration, VAAPS, histopathological examination (HPE)

## Abstract

**Introduction:**

Although carcinomatous degeneration is a rare occurrence, some authors support the need for a histopathological examination after pilonidal cyst excision. Today, minimally invasive techniques are widely spread for the treatment of pilonidal sinus disease but opposed to standard procedures, these techniques could not allow to perform a histopathological examination because of the absence of a specimen. The aim of this two-institutions study is to evaluate whether histopathological examination of the pilonidal sinus excision material can be successfully performed after an endoscopic ablation of the cyst.

**Materials and Methods:**

We identified all consecutive patients from January 2021 to September 2021 with diagnosis of pilonidal sinus disease who underwent Video Assisted Ablation of Pilonidal Sinus (VAAPS) followed by histopathological examination.

**Results:**

A total of 45 patients were included in the study. All patients were Caucasians and aged below 50 years. Nine of them underwent surgery due to recurrence of PSD. No evidence of malignancy was detected in the histopathological examination of the pilonidal sinus sampling material.

**Discussion:**

We were able to send pilonidal sinus sampling material for a histopathological examination in all patients who underwent minimally invasive technique for the treatment of pilonidal sinus disease. No evidence of malignancy was found in any of the 45 samples. Our findings prove that minimally invasive ablation of pilonidal sinus does not preclude histopathological examination of the cysts.

## Introduction

Pilonidal cyst is a benign disease that arise in the sacrococcygeal region with a variable prevalence between 0 and 5%. Pilonidal sinus disease (PSD) progresses with inflammation of the skin and subcutaneous tissue and it may have an acute onset and chronic course with no treatment.

Carcinomatous degeneration of pilonidal cysts is rare and it is probably triggered by chronic inflammatory process. Although the incidence of this condition is estimated at 0.1%, some authors emphasize the need for histopathological examination (1–3).

Today, minimally invasive techniques are widely spread for the treatment of PSD (4–9). These procedures generally involve removing debris and ablation of the granulation tissue under direct vision (10, 11).

Some authors affirm that opposed to a standard procedure, these techniques don't allow to perform histopathological examination because of the absence of a specimen (12).

The aim of this two-institutions study is to evaluate whether histopathological examination of the pilonidal sinus sampling material can be successfully performed after an endoscopic ablation of the cyst.

## Materials and Methods

Utilizing prospectively maintained databases of two centres, we identified all consecutive patients from January 2021 to September 2021 with diagnosis of PSD who underwent Video Assisted Ablation of Pilonidal Sinus (VAAPS) followed by histopathological examination.

As most common low complexity surgical procedures (13–19), all operations were performed in day surgery and under local anaesthesia with 30–60 mL of mepivacaine (Carbosen, 20 mg/mL; Galenica Senese). Patients were placed in prone position with the hips slightly flexed and the buttocks retracted with adhesive tape. The endoscope was inserted through the lower orifice and the sinus cavity was irrigated with a continue flow of saline solution. The debris were removed and mechanical adhesiolysis was performed with the grasping forceps. Then the granulation tissue was completely ablated using a 5-Fr bipolar electrode under direct vision (10, 11, 20) and a complete cleaning of the sinus cavity was performed with the Volkmann spoon. According to current literature, no surgical drain was positioned at the end of the procedure (21).

Pilonidal sinus sampling material harvested by endoscopic ablation and extracted with Volkmann spoon was sent for histopathological examination after surgery in all patients.

Institutional Review Board approval and written informed consent were obtained before review of any patient material.

Patients' characteristics analyzed were: gender, age, BMI, ethnicity, risk factors for pilonidal sinus disease, recurrence of PSD and histopathological examination reports.

Age and BMI were expressed as mean ± standard deviation. Numerical data were expressed as percentage (%).

## Results

Forty-five patients were included in the study, 35 (77.8%) were men and 10 (22.2%) were women. All the patients were Caucasians and they were all aged below 50 years. Mean age was 27.8 ± 9.17 (min = 14 and max = 49) years. Mean BMI was 26.5 ± 4,034 kg/m^2^.

Twelve (26.7%) patients had risk factors for developing PSD: 8 (17.8%) were obese (BMI > 30), 4 (8.9%) had diabetes and hirsutism and 1 (2.2%) had Polycystic Ovary Syndrome (PCOS).

The number of patients that underwent surgery due to recurrence of PSD was determined as 9 (20%), 7 males and 2 females.

Patients' demographics and clinicopathological characteristics are shown in Table 1.

**Table 1:** Demographics and clinicopathological characteristics of the patients.

**Variable**	***N* = 45**
**Gender (%)**	
Male	35 (77.8%)
Female	10 (22.2%)
Age (mean ± SD) (years)	27.8 ± 9.17
BMI (mean ± SD) (kg/m^2^)	26.5 ± 4.34
**Pilonidal sinus disease**	
Primary (%)	36 (80%)
Recurrent (%)	9 (20%)
**Histopathological examination reports**	
Benign (%)	45 (100%)
Malignant (%)	0 (0%)

No evidence of malignancy was detected in the histopathological examination of the pilonidal sinus excision materials in any of the 45 patients as detailed described in Table 2.

**Table 2:** Detailed histopathological results of the patients.

**Histopathology**	**Primary disease**	**Recurrence**	**Total**
	**(*n* = 36)**	**(*n* = 9)**	**(*n* = 45)**
Benign (%)	36 (100%)	9 (100%)	45 (100%)
Pilonidal sinus	24 (66.7%)	0 (0%)	24 (53.3%)
Pilonidal sinus, chronic inflammatory tissue	5 (13.8%)	2 (22.2%)	7 (15.6%)
Abscess	2 (5.6%)	1 (11.1%)	3 (6.7%)
Fibroadipose tissue	2 (5.6%)	0 (0%)	2 (4.4%)
Scar tissue	3 (8.3%)	6 (66.7%)	9 (20%)
Malignant (%)	0 (0%)	0 (0%)	0 (0%)

Of interest, regarding histopathological results of patients with primary disease, pilonidal sinus was reported in 24 (53.3%) of them and fibroadipose tissue was found in two (4.4%) of them. Chronic inflammatory tissue was described in a total of seven (15.6%) patients; of them, five had primary disease and two had recurrence. Abscess was detected in three (6.7%) cases of which one was a recurrence. Ultimately, scar tissue was identified in nine (20%) patients; of them, six had recurrent disease.

## Discussion

Pilonidal cyst is a benign disease that arises in the sacrococcygeal region with a prevalence that varies between 0 and 5%. Pilonidal sinus disease (PSD) progresses with the inflammation of the skin and subcutaneous tissue and may have an acute onset and chronic course with no treatment. The most common complications are related to infectious processes as local cellulitis, abscess formation and recurrence.

According to recent literature, validated surgical techniques for the management of pilonidal sinus are open healing, off-midline primary closure and minimally invasive techniques (sinusectomy and endoscopic approaches).

Surgical management should be individualized and tailored according to the individual PSD. Particularly, minimally invasive techniques are recommended in cases of limited pilonidal cyst with single or multiple pits on the midline while traditional open healing is recommended for complex cases (22). As we know from literature, open healing and midline closure should not be considered effective for their higher frequency of relapse disease, while out-midline primary closure should be preferred (23).

Carcinomatous degeneration of pilonidal cysts is a rare eventuality. Since the first case of malignant degeneration of pilonidal cyst described by Wolff in 1900 (24), the total number of reported cases in lituerature is fewer than 100. The most common histological type is squamous cell carcinoma, occurring in ~90% of cases (25).

The mechanism of malignant degeneration of pilonidal cyst is probably related to chronic inflammatory processes, such as skin ulcers and fistulas, scar tissue (26, 27). Activated inflammatory cells generate free oxygen radicals, which lead to a DNA repair defect associated with malignant transformation in tissues exposed to chronic inflammation (1).

Malignant degeneration of pilonidal sinus is often associated to male gender with a mean age of 50 years and a long period of symptoms with a mean duration of 20 years prior to diagnosis (28, 29). It has also been reported a particular association with immunosuppression and HPV infection (30).

Although carcinomatous degeneration is a rare occurrence, some authors support the need for histopathological examination after pilonidal cyst excision (1–3, 31).

Today, minimally invasive procedures have widely spread for the treatment of PSD (4–8, 32) even in case of complicated and recurrent diseases (32–35).

Opposed to standard surgical intervention, these techniques could not allow to perform histopathological examination because of the absence of a resected specimen (12).

In this study we sent pilonidal sinus harvested material for histopathological examination in all patients who underwent minimally invasive technique for the treatment of PSD.

Being the study conducted during Coronavirus pandemic, histopathological examination reports were notified to all patients by chat or *via* mail. Nowadays, telemedicine is a powerful tool that allows continuity of care, helping surgeons to curb waiting times for consultation and to glean insights into patients' reported outcomes (36).

Reviewing the reports, we have been able to have all 45 histologic responses. Specifically, no evidence of malignancy was found in any of the samples.

Our findings prove that minimally invasive ablation of pilonidal sinus does not preclude histopathological examination of the cysts.

Nevertheless, the great limit of analyzing incisional samples is the inability to diagnose malignant degeneration with assurance.

The other main limitation of this study is the involvement of only two institutions with resulting lack of patients.

## Data Availability Statement

The original contributions presented in the study are included in the article/supplementary material, further inquiries can be directed to the corresponding author.

## Ethics Statement

The studies involving human participants were reviewed and approved by Comitato Etico Università degli Studi di Napoli Federico II. The patients/participants provided their written informed consent to participate in this study.

## Author Contributions

SV, MMi, MD'A, LS, and GD contributed to conception and design of the study. GC, AC, NG, FM, and PS organized the database. MMa analyzed the data. SV and PA wrote the first draft of the manuscript. AD'A, AM, and CS wrote sections of the manuscript. All authors contributed to manuscript revision, read, and approved the submitted version.

## Conflict of Interest

The authors declare that the research was conducted in the absence of any commercial or financial relationships that could be construed as a potential conflict of interest.

## Publisher's Note

All claims expressed in this article are solely those of the authors and do not necessarily represent those of their affiliated organizations, or those of the publisher, the editors and the reviewers. Any product that may be evaluated in this article, or claim that may be made by its manufacturer, is not guaranteed or endorsed by the publisher.
